# Examining the role of MEDLINE as a patient care information resource: an analysis of data from the Value of Libraries study

**DOI:** 10.5195/jmla.2017.87

**Published:** 2017-10-01

**Authors:** Kathel Dunn, Joanne Gard Marshall, Amber L. Wells, Joyce E. B. Backus

## Abstract

**Objective:**

This study analyzed data from a study on the value of libraries to understand the specific role that the MEDLINE database plays in relation to other information resources that are available to health care providers and its role in positively impacting patient care.

**Methods:**

A previous study on the use of health information resources for patient care obtained 16,122 responses from health care providers in 56 hospitals about how providers make decisions affecting patient care and the role of information resources in that process. Respondents indicated resources used in answering a specific clinical question from a list of 19 possible resources, including MEDLINE. Study data were examined using descriptive statistics and regression analysis to determine the number of information resources used and how they were used in combination with one another.

**Results:**

Health care professionals used 3.5 resources, on average, to aid in patient care. The 2 most frequently used resources were journals (print and online) and the MEDLINE database. Using a higher number of information resources was significantly associated with a higher probability of making changes to patient care and avoiding adverse events. MEDLINE was the most likely to be among consulted resources compared to any other information resource other than journals.

**Conclusions:**

MEDLINE is a critical clinical care tool that health care professionals use to avoid adverse events, make changes to patient care, and answer clinical questions.

## INTRODUCTION

Where once physicians found barriers in accessing health information [1], there is now a much greater availability of information resources [2]. This access is essential, given the little time physicians have to research and answer a clinical question. The lack of time available to physicians contrasts with the number of clinical questions raised during patient encounters. Physicians typically have more questions following a clinical encounter than they seek to answer, ranging from 0.07 to 1.85 questions [3]. One literature review found that predictors of physician information-seeking behavior included the existence of an urgent patient problem and the expectation that a clear answer existed [3]. Such clinical questions most often concern primary care and specific patients’ problems and not general medical information. An example of a specific question is: “What is the dose of digoxin for this patient with these problems?” [4]. Thirty percent to 57% of questions lead to information-seeking by physicians [3]. Furthermore, when physicians do take time to search for an answer to a clinical question, they do not tend to spend much time doing the research (typically 2 to 12 minutes [3]).

While physicians still prefer colleagues as their primary source when they first seek an answer to a clinical question [3], when they need information beyond that provided by colleagues, they must often consult multiple sources of information to obtain an answer, ranging from 1.8 resources in an experimental study in which physicians could select any resource of their choosing [5] to as many as 6 resources in a more controlled setting [6].

The previously conducted “Value of Library and Information Services in Patient Care” study (Value of Libraries study) assessed the impact of literature-based resources on patient care. The study surveyed attending physicians, residents, and nurses at 56 participating library sites that served 118 hospitals. Using the critical incident technique, the respondents were asked to base their survey responses on a specific patient care situation in which they had searched for information beyond what was available in the patient record, electronic medical record, or lab results. The respondents provided details of 16,122 information-seeking incidents (5,379 from physicians, 2,123 from residents, and 6,788 from nurses). Similar to previous studies, the Value of Libraries study found that health care providers used an average of 3.5 resources to answer a clinical question [7].

The present study’s research questions were based on a review of the data from the Value of Libraries study. Where the Value of Libraries study reported that using a larger number of information resources was associated with changes in patient care, it did not conduct an in-depth analysis of the impact of an individual resource on patient care or characterize the use of a particular resource relative to other resources. While the Value of Libraries study highlights the role of multiple resources in answering clinical questions, the authors sought to understand what, if any, role MEDLINE plays in information-seeking behaviors in a complex information environment.

## METHODS

We data-mined the results of the Value of Libraries study with a specific focus on questions related to the use of information resources in avoiding adverse events and changing patient care by identifying the use of individual resources and particularly the use of MEDLINE.

Of the 16,122 Value of Libraries survey respondents, 1,578 did not answer the question about the resources that they used to answer their specific clinical questions. Therefore, our analysis was based on the remaining 14,544 survey responses. The sample sizes (n) for the bivariate analyses varied based on the number of people who responded to each cross-section of survey questions. Since most respondents reported using multiple resources to answer their clinical questions, we also explored whether using more resources was associated with reported changes to patient care and avoidance of adverse events.

We created three sets of variables to assess the relationship between the use of information resources and changes to patient care or avoidance of adverse events. The first set of variables indicated which information resources the survey respondents used based on answers to the question, “Recalling the [clinical incident/situation], what resources did you use to search for the information you needed to answer your question?” Respondents could select as many resources as they used from a list of nineteen information resources, with an additional option to select “not sure.” The information sources were, in alphabetical order: books (online/electronic), books (print), CINAHL, Clinical Evidence (BMJ), consumer health resources, DynaMed, eMedicine, ePocrates, Essential Evidence Plus, journals (online/electronic), journals (print), MD Consult, Micromedex, Nursing Reference Center, Ovid MEDLINE, professional association websites, PubMed/MEDLINE, Stat!Ref, and UpToDate. In our analyses, we combined “Ovid MEDLINE” and “PubMed/MEDLINE” into a single category, which we refer to as “MEDLINE,” as both refer to the same content from different sources. We also combined “journals (online/electronic)” and “journals (print)” into a single category and combined “books (online/electronic)” and “books (print)” into a single category.

The second set of variables indicated whether respondents made any changes to patient care as a result of the information that they obtained in their searches based on answers to the question, “Recalling the [clinical incident/situation], did any of the following change in a positive way as a result of the information?” Respondents selected as many changes as applied from a list of eight possible changes: diagnosis, choice of test, choice of drugs, choice of other treatments, length of stay, post-hospital care or treatment, advice given to patient or family, and different handling of the situation.

The third set of variables indicates whether respondents avoided any adverse events as a result of the information that they obtained in their searches based on answers to the survey question, “Recalling the [clinical incident/situation], were any of the following events avoided as a result of the information?” Respondents could select as many events as applied from a list of twelve possible changes: hospital admission, hospital readmission, patient mortality, language or cultural misunderstanding, patient misunderstanding of disease, hospital-acquired infection, surgery, regulatory noncompliance, additional tests or procedures, medication error, adverse drug reaction or interaction, and misdiagnosis.

We used descriptive statistics to examine how frequently each information resource was used alone and in combination with other resources. Because most respondents used more than one information resource, one of which was MEDLINE, our analysis required us to explore various combinations of resources that included MEDLINE, rather than simply focusing on MEDLINE alone.

To examine the extent to which using each information resource or combination of resources was associated with the outcome measures (changes to patient care and avoidance of adverse events), we conducted bivariate analysis using chi-squared tests. Chi-squared values with corresponding *p*-values <0.05 indicate that using the information resource or combination of resources is significantly associated with the outcome measures. To determine whether using more information resources was associated with the outcome measures, we calculated predicted probabilities with 95% confidence intervals derived from logistic regression analyses, a type of predictive analysis used to explain the relationship between 1 dependent binary variable and 1 or more nominal, ordinal, or ratio-level variables. Predicted probabilities with *p*-values <0.05 indicate that the number of resources used is significantly associated with the outcome measures.

## RESULTS

**Figure 1:**
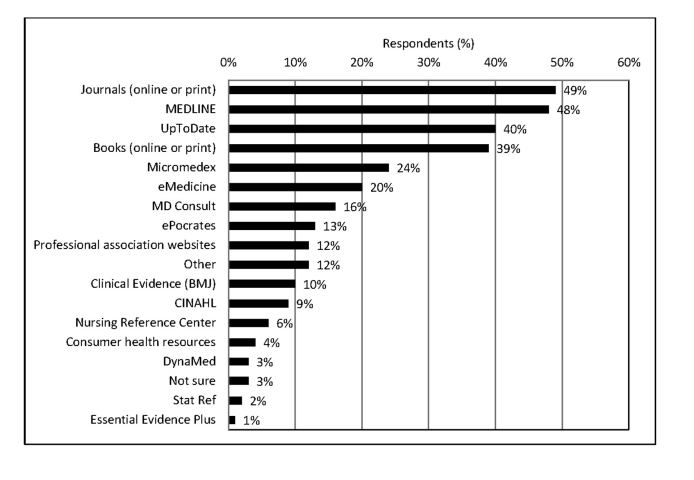
Information resources used (n=14,544)

Most respondents (75%) used more than 1 information resource to answer their clinical questions, with an average of 3.5 resources used (range, 1 to 19). Journals (online or print) and MEDLINE were the top 2 information resources that respondents used (Figure 1).

Considering the well-established finding that clinicians use multiple information sources [5, 6, 8], we examined how often MEDLINE was used in combination with other information sources (Table 1). We found that MEDLINE was used in all of the top ten most frequently used combinations of information resources. As the number of resources used to answer the clinical question increased, MEDLINE was more likely to be included in the top ten most frequently used combinations of resources. That is, MEDLINE appeared in two of the top ten combinations when only two resources were used, in nine of the top ten combinations when three or four resources were used, and all of the top ten combinations when five or more resources were used.

**Table 1:** Number of times MEDLINE appeared in the top 10 most frequent combinations of information resources used (n=14,544)

**Number of resources used**	**Number of times MEDLINE was used**	**n**
1	1	3,588
2	2	2,630
3	9	2,456
4	9	1,938
5	10	1,354
6	10	919
7+	10	1,659

Next, we examined the extent to which those who used MEDLINE also reported changes in patient care and avoided adverse events as a result of the information they obtained in their searches. The percent of respondents who used MEDLINE as one of the resources in their searches among those who reported a change made to patient care is shown in Table 2. We found that use of MEDLINE was significantly associated with all eight identified changes to patient care. MEDLINE was also one of the most commonly consulted resources when specific patient care changes were made; that is, all eight of the possible changes to patient care, MEDLINE was the second most frequently used resource after journals.

**Table 2:**
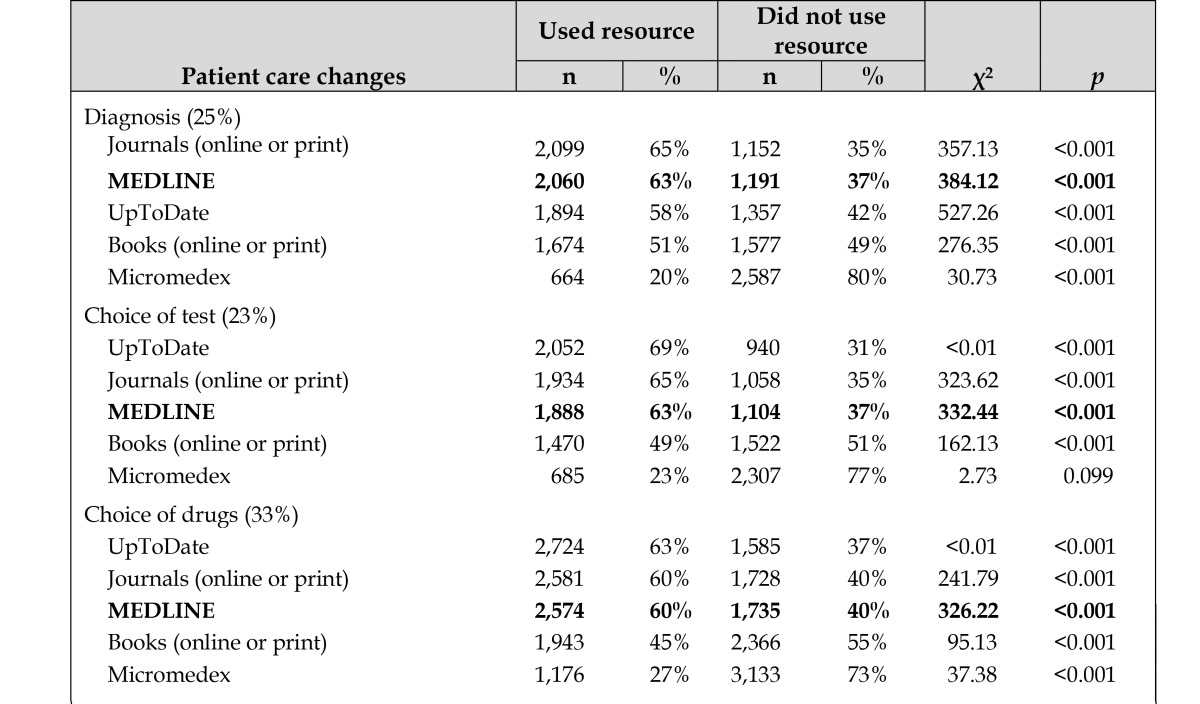
Percent of respondents who used each information resource among those who made changes to patient care as a result of the information obtained in their searches

**Patient care changes**	**Used resource**		**Did not use resource**		**χ^2^**	***p***

	**n**	**%**	**n**	**%**		
Diagnosis (25%)						
Journals (online or print)	2,099	65%	1,152	35%	357.13	<0.001
**MEDLINE**	**2,060**	**63%**	**1,191**	**37%**	**384.12**	**<0.001**
UpToDate	1,894	58%	1,357	42%	527.26	<0.001
Books (online or print)	1,674	51%	1,577	49%	276.35	<0.001
Micromedex	664	20%	2,587	80%	30.73	<0.001
Choice of test (23%)						
UpToDate	2,052	69%	940	31%	<0.01	<0.001
Journals (online or print)	1,934	65%	1,058	35%	323.62	<0.001
**MEDLINE**	**1,888**	**63%**	**1,104**	**37%**	**332.44**	**<0.001**
Books (online or print)	1,470	49%	1,522	51%	162.13	<0.001
Micromedex	685	23%	2,307	77%	2.73	0.099
Choice of drugs (33%)						
UpToDate	2,724	63%	1,585	37%	<0.01	<0.001
Journals (online or print)	2,581	60%	1,728	40%	241.79	<0.001
**MEDLINE**	**2,574**	**60%**	**1,735**	**40%**	**326.22**	**<0.001**
Books (online or print)	1,943	45%	2,366	55%	95.13	<0.001
Micromedex	1,176	27%	3,133	73%	37.38	<0.001
Choice of other treatments (31%)						
Journals (online or print)	2,702	66%	1,399	34%	587.58	<0.001
**MEDLINE**	**2,604**	**63%**	**1,497**	**37%**	**539.47**	**<0.001**
UpToDate	2,204	54%	1,897	46%	395.51	<0.001
Books (online or print)	2,019	49%	2,082	51%	254.52	<0.001
Micromedex	926	23%	3,175	77%	6.85	0.009
Length of stay (7%)						
Journals (online or print)	589	63%	353	37%	61.81	<0.001
**MEDLINE**	**553**	**59%**	**389**	**41%**	**42.64**	**<0.001**
Books (online or print)	499	53%	443	47%	81.44	<0.001
UpToDate	493	52%	449	48%	53.22	<0.001
Micromedex	271	29%	671	71%	12.49	<0.001
Post-hospital care or treatment (12%)						
Journals (online or print)	1,028	63%	598	37%	126.13	<0.001
**MEDLINE**	**962**	**59%**	**664**	**41%**	**85.10**	**<0.001**
Books (online or print)	830	51%	796	49%	110.30	<0.001
UpToDate	763	47%	863	53%	26.29	<0.001
Micromedex	480	30%	1,146	70%	30.65	<0.001
Advice given to patient or family (48%)						
Journals (online or print)	3,402	54%	2,849	46%	85.55	<0.001
**MEDLINE**	**3,250**	52%	**3,001**	48%	**59.49**	**<0.001**
UpToDate	2,885	46%	3,366	54%	127.21	<0.001
Books (online or print)	2,612	42%	3,639	58%	34.86	<0.001
Micromedex	1,774	28%	4,477	72%	123.55	<0.001
Different handling of situation (21%)						
Journals (online or print)	1,658	60%	1,110	40%	132.29	<0.001
**MEDLINE**	**1,608**	**58%**	**1,160**	**42%**	**130.24**	**<0.001**
Books (online or print)	1,317	48%	1,451	52%	104.69	<0.001
UpToDate	1,249	45%	1,519	55%	23.80	<0.001
Micromedex	779	28%	1,989	72%	32.51	<0.001

* Note: Numbers in parentheses indicate the percent of respondents who reported having made changes to patient care regardless of information resource used (n=13,151).

The percent of respondents who used MEDLINE as one of the resources in their searches among those who reported avoiding adverse events is shown in Table 3. We found that use of MEDLINE was significantly associated with avoiding all twelve of the adverse events. It was also one of the most commonly consulted resources when specific adverse events were avoided; MEDLINE was the first or second most frequently used resource, after journals, in ten of the twelve possible adverse events.

**Table 3:**
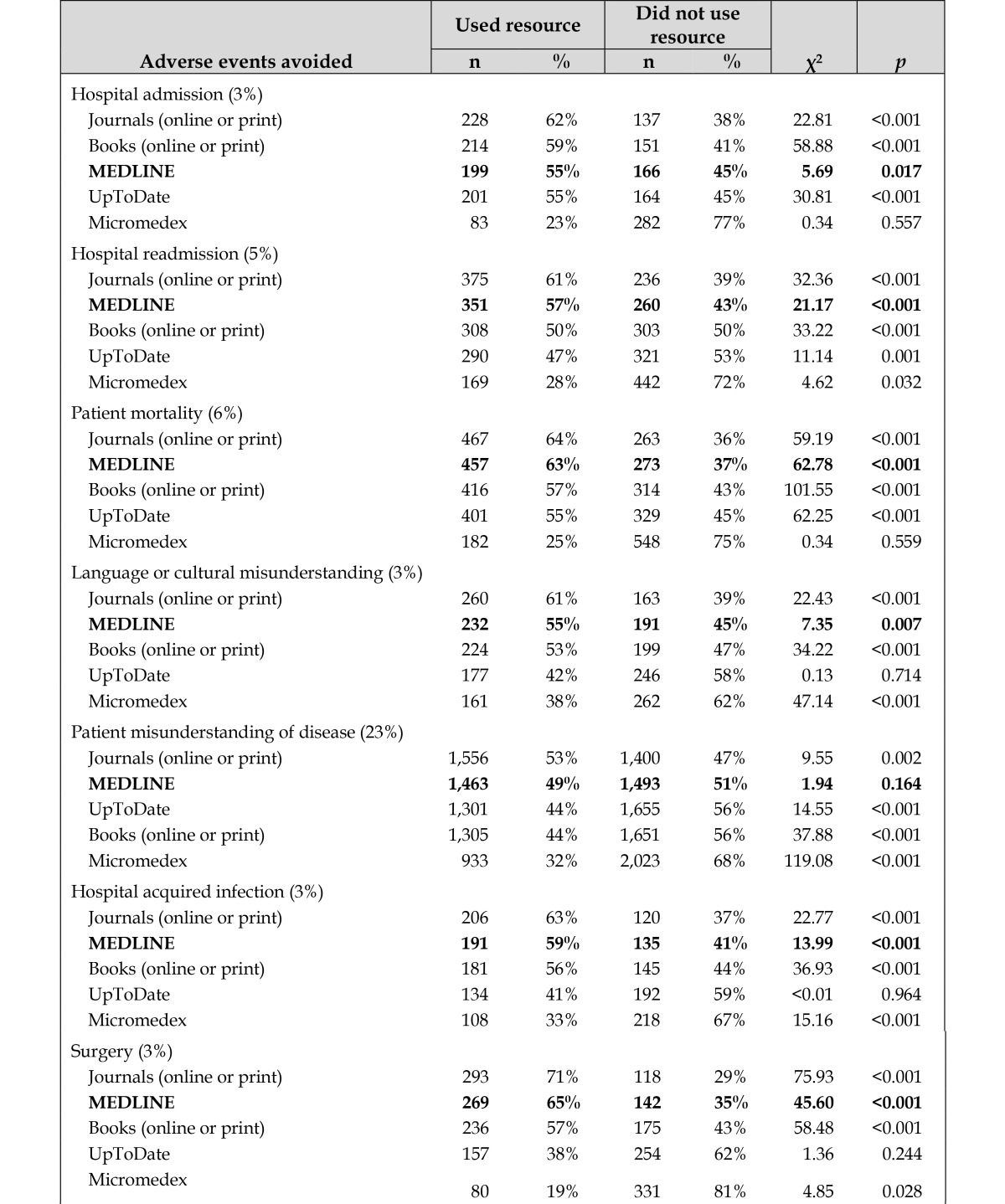
Percent of respondents who used each information resource among those who avoided adverse events as a result of the information obtained in their searches

**Adverse events avoided**	**Used resource**		**Did not use resource**		**χ^2^**	***p***

	**n**	**%**	**n**	**%**		
Hospital admission (3%)						
Journals (online or print)	228	62%	137	38%	22.81	<0.001
Books (online or print)	214	59%	151	41%	58.88	<0.001
**MEDLINE**	**199**	**55%**	**166**	**45%**	**5.69**	**0.017**
UpToDate	201	55%	164	45%	30.81	<0.001
Micromedex	83	23%	282	77%	0.34	0.557
Hospital readmission (5%)						
Journals (online or print)	375	61%	236	39%	32.36	<0.001
**MEDLINE**	**351**	**57%**	**260**	**43%**	**21.17**	**<0.001**
Books (online or print)	308	50%	303	50%	33.22	<0.001
UpToDate	290	47%	321	53%	11.14	0.001
Micromedex	169	28%	442	72%	4.62	0.032
Patient mortality (6%)						
Journals (online or print)	467	64%	263	36%	59.19	<0.001
**MEDLINE**	**457**	**63%**	**273**	**37%**	**62.78**	**<0.001**
Books (online or print)	416	57%	314	43%	101.55	<0.001
UpToDate	401	55%	329	45%	62.25	<0.001
Micromedex	182	25%	548	75%	0.34	0.559
Language or cultural misunderstanding (3%)						
Journals (online or print)	260	61%	163	39%	22.43	<0.001
**MEDLINE**	**232**	**55%**	**191**	**45%**	**7.35**	**0.007**
Books (online or print)	224	53%	199	47%	34.22	<0.001
UpToDate	177	42%	246	58%	0.13	0.714
Micromedex	161	38%	262	62%	47.14	<0.001
Patient misunderstanding of disease (23%)						
Journals (online or print)	1,556	53%	1,400	47%	9.55	0.002
**MEDLINE**	**1,463**	**49%**	**1,493**	**51%**	**1.94**	**0.164**
UpToDate	1,301	44%	1,655	56%	14.55	<0.001
Books (online or print)	1,305	44%	1,651	56%	37.88	<0.001
Micromedex	933	32%	2,023	68%	119.08	<0.001
Hospital acquired infection (3%)						
Journals (online or print)	206	63%	120	37%	22.77	<0.001
**MEDLINE**	**191**	**59%**	**135**	**41%**	**13.99**	**<0.001**
Books (online or print)	181	56%	145	44%	36.93	<0.001
UpToDate	134	41%	192	59%	<0.01	0.964
Micromedex	108	33%	218	67%	15.16	<0.001
Surgery (3%)						
Journals (online or print)	293	71%	118	29%	75.93	<0.001
**MEDLINE**	**269**	**65%**	**142**	**35%**	**45.60**	**<0.001**
Books (online or print)	236	57%	175	43%	58.48	<0.001
UpToDate	157	38%	254	62%	1.36	0.244
Micromedex	80	19%	331	81%	4.85	0.028
Regulatory noncompliance (2%)						
Journals (online or print)	202	64%	112	36%	25.91	<0.001
**MEDLINE**	**198**	**63%**	**116**	**37%**	**27.80**	**<0.001**
Books (online or print)	162	52%	152	48%	20.41	<0.001
UpToDate	131	42%	183	58%	0.07	0.788
Micromedex	113	36%	201	64%	25.19	<0.001
Additional tests or procedures (19%)						
Journals (online or print)	1,637	65%	877	35%	279.97	<0.001
**MEDLINE**	**1,573**	**63%**	**941**	**37%**	**252.14**	**<0.001**
UpToDate	1,571	62%	943	38%	597.14	<0.001
Books (online or print)	1,206	48%	1,308	52%	98.58	<0.001
Micromedex	532	21%	1,982	79%	14.10	<0.001
Medication error (12%)						
UpToDate	855	58%	630	42%	191.03	<0.001
Journals (online or print)	821	55%	664	45%	17.75	<0.001
**MEDLINE**	**816**	**55%**	**669**	**45%**	**29.10**	**<0.001**
Books (online or print)	705	47%	780	53%	47.09	<0.001
Micromedex	534	36%	951	64%	130.74	<0.001
Misdiagnosis (13%)						
Journals (online or print)	1,135	66%	593	34%	192.72	<0.001
**MEDLINE**	**1,123**	**65%**	**605**	**35%**	**220.68**	**<0.001**
UpToDate	1,092	63%	636	37%	406.98	<0.001
Books (online or print)	873	51%	855	49%	105.44	<0.001
Micromedex	313	18%	1,415	82%	38.30	<0.001
Adverse drug reaction or interaction (13%)						
**MEDLINE**	**1,012**	**61%**	**642**	**39%**	**124.79**	**<0.001**
Journals (online or print)	991	60%	663	40%	72.47	<0.001
UpToDate	926	56%	728	44%	176.55	<0.001
Books (online or print)	816	49%	838	51%	80.20	<0.001
Micromedex	653	39%	1,001	61%	247.95	<0.001

* Note: Numbers in parentheses indicate the percent of respondents who reported having avoided adverse events regardless of information resource used (n=12,903).

**Figure 2:**
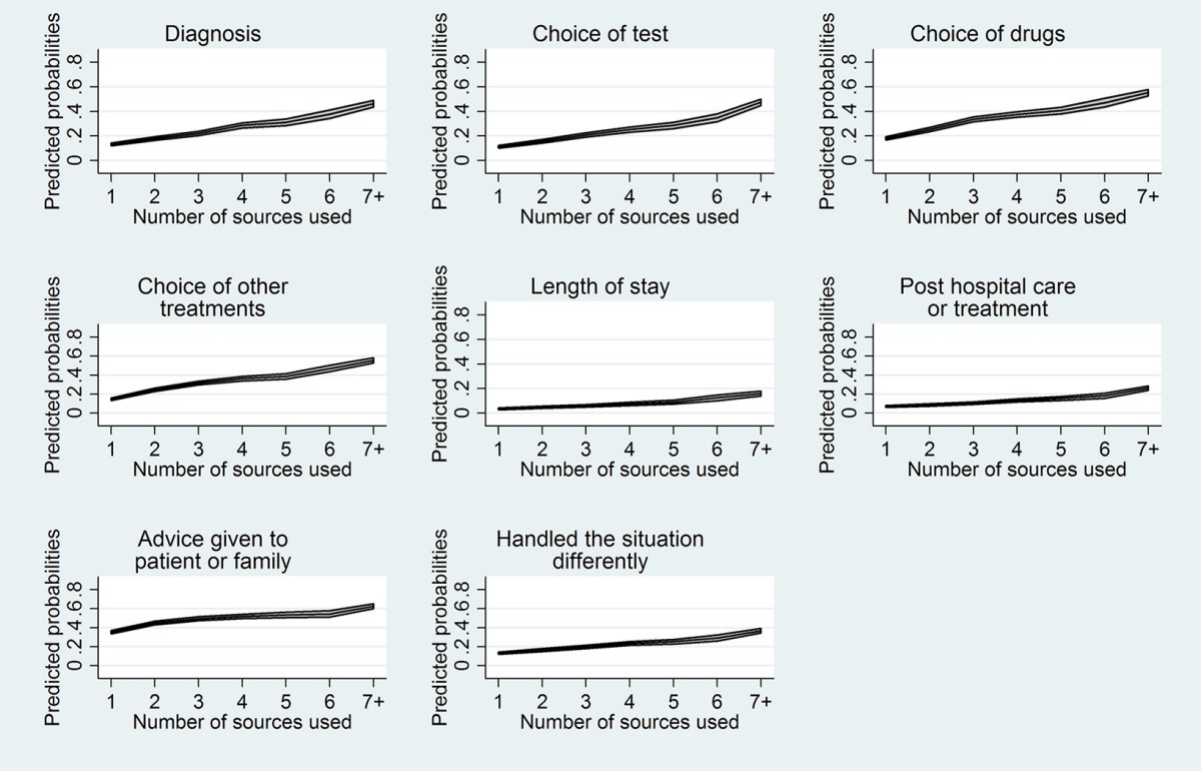
Predicted probabilities for changes made to patient care (with 95% confidence intervals), depending on the number of information resources used

**Figure 3:**
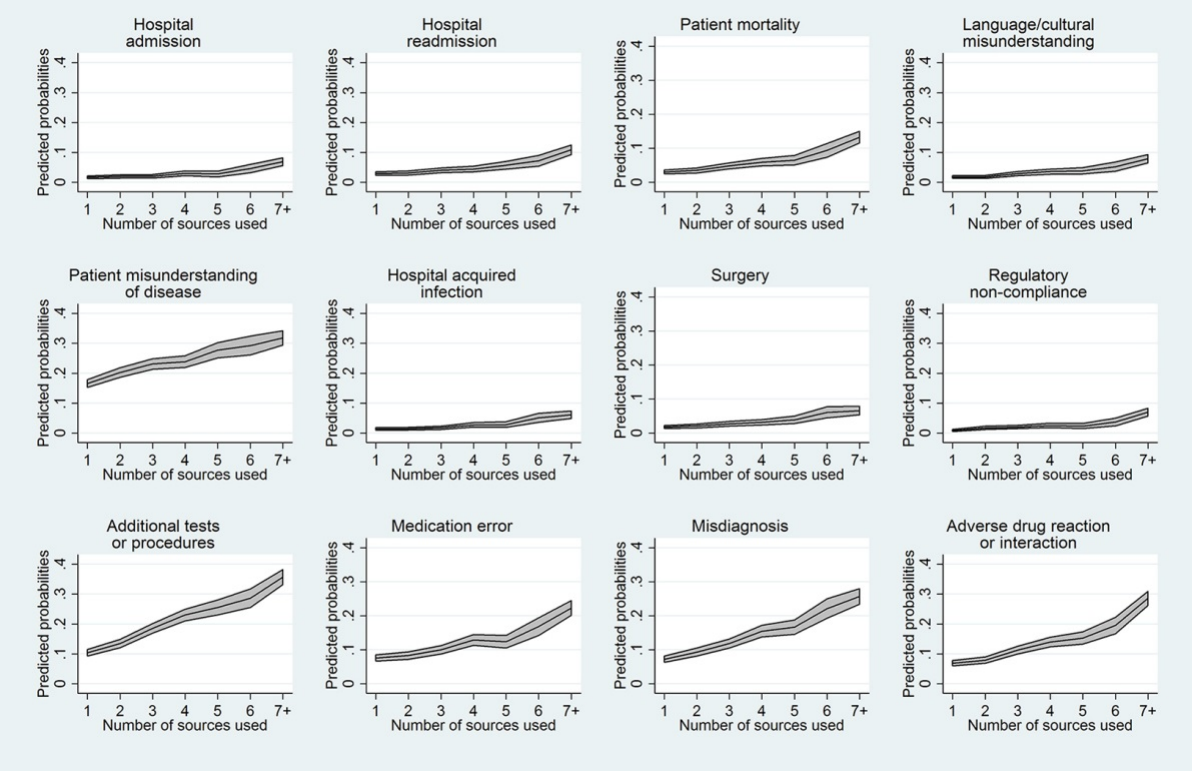
Predicted probabilities for adverse events avoided (with 95% confidence intervals), depending on the number of information resources used

Lastly, we examined whether using more information resources was significantly associated with changes in patient care. We found that using a larger number of information resources to answer a clinical question was significantly associated with greater probabilities of making all 8 possible changes to patient care (*p*<0.05, Figure 2). Likewise, using a larger number of information resources was associated with greater probabilities of avoiding all 12 possible adverse events (*p*<0.05, Figure 3).

## DISCUSSION

We found that using multiple resources is associated with making more changes to patient care and avoiding adverse events. Given the tendency of respondents to consult several resources to answer a clinical question, the clinical questions reported by these respondents are likely not fact based but rather are complex and require the synthesis of information across multiple resources. Thus, to answer their clinical questions, health care providers are more likely to need a well-curated collection of information resources, such as that provided by a library, rather than a single, standalone resource.

MEDLINE is directly searchable from the National Library of Medicine (NLM) as a subset of the PubMed database as well as through several other search services that license the data. The combination of MEDLINE and the additional features provided by PubMed, including citations that may have links to full-text articles or manuscripts in PubMed Central, have made this the premier biomedical literature database in the world, currently comprising over 25 million citations. The combined PubMed/MEDLINE database has been available online since 1966 and continues to be one of the most widely accessible resources for scientists and health care providers.

PubMed/MEDLINE has been heavily studied, with researchers examining its utility in patient care [9] and improved decision making [10, 11]. Previous research has either focused on the use of MEDLINE as a single resource or has compared the use of MEDLINE with one other resource [12, 13]. However, the current information-rich environment makes MEDLINE one of many resources found on the web [6, 8], in clinical information portals [14], and in suites of resources available from libraries [15]. Other literature-based resources, particularly point-of-care tools, have emerged in the last decade and have played a prominent role in health care providers’ learning and decision making [16].

Data from this study add to the body of literature about MEDLINE, demonstrating that MEDLINE is an effective component in providing answers to clinical questions, as it appears more frequently than any other resource with the exception of journals (print and online), and in effectively changing patient care. MEDLINE often functions as a “pointer” to journals and is the basis from which point-of-care tools and other secondary resources develop the information used in their products. Thus, the extent to which the use of MEDLINE and journals can be fully separated is open to question, because MEDLINE itself includes abstracts as well as many links to full-text journal articles in PubMed Central. A similar situation may exist between the synthesized resources found in UpToDate and the primary literature published in journals. Although our data point toward the use of and need for multiple resources in answering clinical questions, the high use of journals (print and online), MEDLINE, and UpToDate potentially indicates that the resources are used independently of each other, with the full text of journal articles, abstracts in MEDLINE, or the synthesized information in UpToDate providing an answer to a clinical question.

Our findings may encourage other researchers to undertake more detailed studies on the use of multiple information resources. The archiving of research datasets in library and information science is in its infancy, and library and information researchers may be less familiar with secondary data analysis than researchers in other fields, particularly the social sciences. Thus, our study illustrates the potential usefulness of secondary data analysis and may prompt other researchers to consider archiving their data and making the data available to others. There are challenges in reusing data, including the limitations of the original study [7]. Significantly, the Value of Libraries study originally collected data to examine all resources used to effect changes in patient care, whereas the current study examined the roles of individual resources, notably MEDLINE, in having an impact on patient care.

Of particular interest to health sciences librarians is our finding that clinicians who used more information resources reported making more changes to patient care and avoided more adverse events as a result of the information obtained in their searches. Our finding that providers used an average of 3.5 information resources in their searches may have been affected by our critical incident methodology. Because health professionals were asked to base their answers to the survey on one clinical situation, the respondents might have selected a situation in which a more complex search was required.

In any case, our results suggest that more complex searches are being done and that health professionals are using a variety of different resources rather than a single resource. By providing access to a wide range of electronic and print information resources, libraries play an essential role in improving the quality of health care and evidence-based practice.

## AVAILABILITY OF DATA AND MATERIALS

The data set supporting the conclusions of this article is available in the Odum Institute Archive Dataverse.
